# Case of Gun-Shot Wound of Kidney—Recovery

**Published:** 1864-05

**Authors:** R. J. Perry

**Affiliations:** Assistant Surgeon P. A. C. S.


ART. VI.—*Case of Gun-Shot Wound of Kidney.—Recovery.* By R. J. PERRY, Assistant Surgeon P. A. C. S.

The records of military surgery show that gun-shot wounds of the kidney are almost always fatal, and being so considered, the unfortunate victim is too often left to his fate without proper attention. The following case presents several points of unusual novelty and interest, and teaches the important lesson that the surgeon should never abandon, as hopeless, any case of injury, however unpromising it may seem. Patients do occasionally recover from wounds of the kidney as well as from lumbar abscesses, caused by renal calculi, and should therefore always be treated with proper care throughout.

Lieut. A., 2d Tennessee infantry, in perfect health, of robust constitution and abstemious habits, was wounded in battle of Shiloh, on April 6th, 1862, by a Minie ball entering immediately below the heart, and passing out through the upper portion of the left kidney. There was considerable hemorrhage causing excessive prostration. In this condition he was captured by the enemy and removed to Pittsburg landing on Tennessee river, several miles distant from the battle-field, where he remained for six days without any attention, not even the removal of his bloody clothing, or dressing of his wounds. He was then placed upon a transport and conveyed to Louisville, Kentucky, and sent to hospital for treatment.

During the month of July following, whilst his wounds were still discharging profusely, he was attacked with typhoid fever, and a large abscess formed in the lower part of abdomen, about one inch to the left of the linea alba, which caused great pain. The second or third week in August he was removed from Louisville to Camp Chase, by way of Cincinnati. Several days after his arrival at Camp Chase, very much enervated from the prolonged attack of fever, the abscess above referred to opened outwardly, and discharged an immense quantity of dark sanious fluid mixed with urine.—This greatly alarmed him, and the extreme mental anxiety added to his fearful nervous prostration, came near proving too formidable for the unfortunate victim, but all of these difficulties were combatted by a good constitution and the inflexible determination of a veteran soldier to such a degree, that when an exchange of prisoners was effected, he was able to proceed to Vicksburg, Miss., where he was released about the first of October.

He commenced his journey homeward, (Lynchburg, Va.,) travelling only during the day, resting at night, suffering much from his wounds and abscess, which still continued to discharge an admixture of unhealthy pus and urine. In about two weeks he reached Knoxville, Tenn., (at which place I was then on duty,) manifesting symptoms of very great nervous prostration. The second day after his arrival at Knoxville I was called to see him, at the house of his sister, at 9 o'clock, A. M., and found him with a severe chill, followed by high febrile reaction. On examination I found the anterior wound entirely healed and cicatrized—the posterior wound and abscess very irritable, manifesting no disposition to heal, and both discharging, though not profusely, a thin sanious fluid, mixed with urine. He complained of severe, excruciating pains in lumbar region, passing but little urine through the urethra —secretions generally deranged. I ordered warm stimulating poultices to wound and abscess, and administered one grain of extract of hyoscyamus.

I visited him again at 4 o'clock, P. M., found him restless, looking pale, anxious and alarmed, pulse irritable and frequent, administered anodyne for the night. I saw him the succeeding morning at 9 o'clock; rested rather comfortably during the night, still suffering from pains in lumbar region, but much more composed, pulse regular but frequent—continued warm applications to wound and abscess, and anodynes to relieve pain. For several days subsequent he was annoyed with rigors simulating intermittent fever, but which gradually subsided, leaving him much debilitated and troubled with night-sweats, which were overcome by the use of elixir vitriol, tannin, and sponging with stimulating lotions. I then placed him upon nutritious diet and tonics, such as iron, tincture of bark and quinine. The discharge of urine and unhealthy pus continued for some sixteen or eighteen days, when the discharge of urine ceased, and the pus became more laudable. Simple lint and sweet oil dressings were then substituted for the warm applications.

The second or third week in November the wound was almost entirely healed, with but slight discharge, and about the 15th of December he resumed his journey to Lynchburg, and in a short time was entirely restored, with some little impairment of general health. I met Lieut. A. in October, 1863, in perfect health, with the exception that upon too free exercise or exposure he was annoyed with some uneasiness and pain in lumbar region.

